# Breaking the unbreakable: A paediatric case of dolutegravir resistance from KwaZulu-Natal

**DOI:** 10.4102/sajhivmed.v24i1.1458

**Published:** 2023-05-23

**Authors:** Sibongiseni Malinga, Aabida Khan, Moherndran Archary

**Affiliations:** 1 Department of Paediatrics, King Edward VIII Hospital, Durban, South Africa; 2 Department of Virology, Inkosi Albert Luthuli Central Hospital, National Health Laboratory Service (NHLS), Durban, South Africa; 3 School of Laboratory Medicine and Medical Sciences, University of KwaZulu-Natal, Durban, South Africa; 4 Department of Paediatrics and Child Health, Faculty of Health Sciences, University of KwaZulu-Natal, Durban, South Africa

**Keywords:** paediatrics, dolutegravir, DTG, resistance, HIV, adolescence

## Abstract

We report a case of dolutegravir resistance in KwaZulu-Natal in a 13-year-old male two years after starting dolutegravir. Resistance most likely developed due to poor adherence as a result of psychosocial issues. This case highlights the importance of the role of the family unit in impacting adherence and close monitoring of treatment-experienced patients with virologic failure following switching to dolutegravir-based regimens.

## What this study adds

Numerous challenges affect HIV-infected children and adolescents, impacting adherence, as highlighted in this case. The benefit of HIV drug resistance genotyping to guide the selection of a suitable regimen was shown – there is a need for evidence-based guidelines of indications for resistance testing on dolutegravir.

## Introduction

South Africa transitioned to dolutegravir (DTG)-based antiretroviral therapy (ART) from 2019.^1^ Various studies have demonstrated that DTG-based ART is highly effective at achieving viral suppression in both ART-naïve and -experienced individuals. Dolutegravir has better tolerability, higher genetic barrier to resistance and is available in a fixed-dose combination of tenofovir (TDF), lamivudine (3TC), and DTG for adults and adolescents ≥ 35 kg.^1^ In South Africa, DTG is currently available as a 50 mg tablet for children ≥ 20 kg.^1^ We present the first paediatric case of DTG resistance identified in a treatment-experienced integrase strand inhibitor (InSTI)-naïve adolescent in KwaZulu-Natal.

## Case

Our index case is a 13-year-old male from a rural area in who acquired HIV through vertical transmission. The mother tested negative during antenatal care, but she was not tested at delivery. He was diagnosed with HIV and pulmonary tuberculosis in 2010, started tuberculosis treatment and later on ART. As per the national guidelines, abacavir, 3TC and ritonavir-boosted lopinavir (LPV/r) syrups were initiated^1^ but it is unclear if he received ritonavir super-boosting during his tuberculosis treatment to overcome the inducing effect of rifampicin. He was diagnosed with tuberculosis again in 2013, again without documentation that LPV/r was super-boosted with ritonavir during treatment. Since ART initiation, he has never been virally suppressed, despite ongoing enhanced adherence support.

**FIGURE 1:**
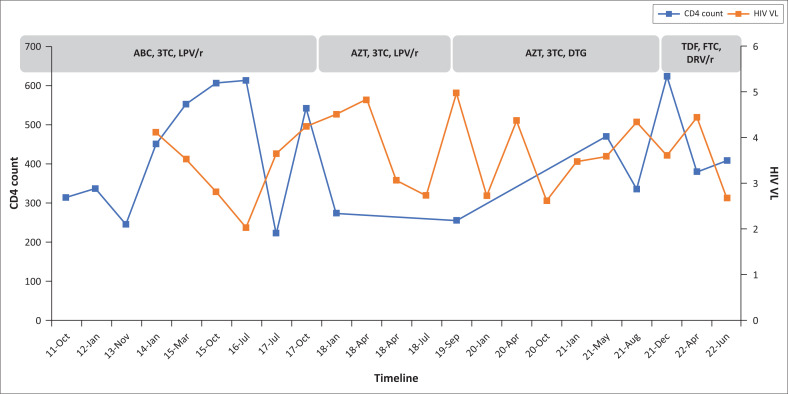
HIV viral load and CD4 count history. ABC, abacavir; 3TC, lamivudine; LPV/r, ritonavir-boosted lopinavir; AZT, zidovudine; DTG, dolutegravir; TDF, tenofovir; FTC, emtricitabine; DRV/r, ritonavir-boosted darunavir; VL, viral load.

Figure 1 shows the HIV viral load (VL) and CD4 count timeline after ART initiation with no evidence of viral suppression defined as < 50 copies/mL.^1^ HIV VL is in log_10_ copies/mL (right vertical axis) and CD4 count is in cells/µL (left vertical axis). HIV VL ≥ 1000 copies/mL (≥ 3 log_10_ copies/mL) is defined as virological failure.^1^

In 2018 HIV drug resistance (DR) genotyping was performed, showing only M184V mutation with no protease resistance mutations, which suggested suboptimal adherence. He was switched to zidovudine (AZT) and 3TC, and was started on LPV/r paediatric tablets. The patient received continuous enhanced adherence counselling by the attending doctor and via the social worker. In January 2020, LPV/r was switched to DTG in an attempt to simplify the regimen and decrease the pill burden; however, he remained on AZT/3TC as he weighed 28 kg. The VL at the time of the switch was 529 copies/mL and hence a HIV DR test was not performed. However, he remained unsuppressed and admitted to often forgetting his medication as he lacked a treatment supporter.

There were major social issues impacting adherence; he is the product of rape by his mother’s older cousin. Being a teen mom, and not having disclosed the rape issue to her mother proved to have affected the patient’s mother, as well as her ability to provide optimum care for her child. She was the primary caregiver living with the maternal grandmother and was responsible for bringing him for his hospital appointments, but would sometimes not be available to bring him, and occasionally the patient’s biological father would accompany the patient to hospital but identified himself as the patient’s uncle. In 2017, the father became ill, and the grandmother (not knowing the issues around the rape) took him into their home, resulting in family conflict and missed hospital appointments. In 2019, both the patient’s biological parents died due to AIDS-related complications. Upon discovering for the first time at her daughter’s funeral that her nephew had raped her daughter and infected her with HIV, the patient’s grandmother struggled to accept the situation. As a result, the grandmother avoided involving herself in the healthcare of her grandson including supervising him when taking treatment, or accompanying him to hospital, making addressing the adherence issues and disclosure difficult, requiring a home visit by the social workers.

Due to poor adherence and patient not suppressing for two years on the DTG-based regimen, despite enhanced adherence counselling, HIV DR was done in April 2022 which showed M184V again and the development of InSTI mutations (major T66TI, G118R, E138K, minor M50I), conferring high-level resistance to DTG (refer to Table 1).

**TABLE 1:** HIV drug resistance genotyping results April 2022.

Drug class	Drug resistance mutations	Drug name	Resistance level	Mutation score
PI major	None	ATV	Susceptible	0
		DRV	Susceptible	0
PI accessory	L23LI	LPV	Susceptible	0
NRTI	M184V	ABC	Low-level resistance	15
		AZT	Susceptible	-10
		3TC	High-level resistance	60
		FTC	High-level resistance	60
		TDF	Susceptible	-10
NNRTI	None	EFV	Susceptible	0
		RPV	Susceptible	0
		ETR	Susceptible	0
InSTI major	T66TI, G118R, E138K	DTG	High-level resistance	75
InSTI minor	M50I	RAL	High-level resistance	100
		CAB	High-level resistance	90
		BIC	Intermediate resistance	55

3TC, lamivudine; ABC, abacavir; ATV, atazanavir; AZT, zidovudine; BIC, bictegravir; CAB, cabotegravir; DRV, darunavir; DTG, dolutegravir; EFV, efavirenz; ETR, etravirine; FTC, emtricitabine; InSTI, integrase strand transfer inhibitor; LPV, lopinavir; NNRTI, non-nucleoside reverse transcriptase inhibitor; NRTI, nucleoside reverse transcriptase inhibitor; NVP, nevirapine; PI, protease inhibitor; RAL, raltegravir; RPV, rilpivirine; TDF, tenofovir.

The third-line regimen commenced in May 2022 was TDF, emtricitabine and ritonavir-boosted darunavir once daily. The viral load done after one month was 473 copies/mL with a 2 log_10_ drop.

## Discussion

This case report highlights the complexities and psycho-social factors impacting the management of HIV in children and adolescents. The paediatric population has unique challenges such as disclosure, caregiver issues, challenges with understanding and acceptance of the disease, limited drug options and child-friendly formulations.

In this case, family issues with a lack of supervision resulted in suboptimal adherence, which resulted in the emergence of DTG resistance. Due to his weight of 28 kg in 2020, he was not eligible for a simple once-daily fixed-dose combination of TDF, 3TC and DTG, which may have improved adherence and potentially prevented DTG resistance.

Most clinical trials have evaluated DTG in adults. The ODYSSEY (Once-daily DTG based ART in Young people vs. Standard therapy) trial done in children and adolescents found that DTG was superior to standard care with lower rates of treatment failure.^2^ None of the participants on first-line DTG-based ART with treatment failure had DTG resistance mutations, while 4/22 (18%) participants with virologic failure on second-line DTG-based ART had DTG resistance.^2^ Similarly, in the IMPAACT P1093 study that assessed DTG in treatment-experienced children and adolescents, 8/36 (22%) participants with virologic failure had resistance to DTG.^3^

The most common mutations selected by DTG in InSTI-naïve patients are reported to be R263K and G118R.^4^ R263K reduces DTG susceptibility two-fold, conferring intermediate resistance, while G118R reduces DTG susceptibility by > 5-fold, also conferring intermediate resistance.^4^ Other resistance mutations selected by DTG in InSTI-naïve patients include E138K/T, N155H, Q148K, S230R, T66I and H51Y.^4^ Accumulation of mutations further reduces DTG susceptibility. These reported mutations are similar to those found in our case.

In sub-Saharan Africa, real-world cohorts have shown high viral suppression rates in countries that have transitioned to DTG. Although DTG is hailed as the silver bullet, it must still be recognised that treatment failure and resistance, especially in treatment-experienced patients with suboptimal adherence, can occur. The potential factors contributing to DTG resistance include poor adherence, DTG monotherapy, advanced HIV and opportunistic infections.^5^

An evaluation of the ADVANCE (dolutegravir plus two different prodrugs of tenofovir to treat HIV) and NAMSAL (new antiretroviral and monitoring strategies in HIV-infected adults in low-income countries) studies highlighted the impact of social and demographic factors on adherence even on DTG-based ART with younger age associated with reduced adherence.^6^ The family unit plays an important role in supporting children and adolescents. Decentralisation of care and other avenues of support such as community based family or youth clubs can also help to improve adherence.

The value of VL monitoring and DR genotyping is crucial in detecting cases of resistance. With increasing transitioning to DTG, population surveillance for DTG resistance must be implemented. There is also a need for the availability of a wide repertoire of drug options that are effective, tolerable and cost-effective with child-friendly formulations.

## Conclusion

This report describes the first case of DTG resistance identified in the paediatric population in KwaZulu-Natal in a treatment-experienced adolescent. It highlights the impact of family and psychosocial issues on adherence, issues affecting adherence in children and paediatric population (including availability of a caregiver to supervise drinking of medications, coming for hospital appointments, poorly tolerated regimens and the lack of availability of simplified treatment options [fixed-dose combination] for paediatric populations), and the need to closely monitor treatment-experienced patients with virologic failure, even on DTG-based regimens. The availability of the abacavir/3TC fixed-dose combination and DTG dispersible tablet will help address some of these issues and improve adherence.

## References

[1] South African National Department of Health. National consolidated guidelines for the management of HIV in adults, adolescents, children and infants and prevention of mother-to-child transmission 2019 [homepage on the Internet]. February 2020. Available from: https://www.knowledgehub.org.za/e-library

[2] Turkova A, White E, Mujuru HA, et al. Dolutegravir as first or second-line treatment for HIV-1 infection in children. N Engl J Med. 2021 Dec 30;385(27):2531–2543. 10.1056/NEJMoa2108793 34965338 PMC7614690

[3] Vavro C, Ruel T, Wiznia A, et al. Emergence of resistance in HIV-1 integrase with dolutegravir treatment in a pediatric population from the IMPAACT P1093 study. Antimicrob Agents Chemother. 2021 Oct 25;66(1):e0164521. 10.1128/AAC.01645-21 34694878 PMC8765298

[4] Rhee SY, Grant PM, Tzou PL, et al. A systematic review of the genetic mechanisms of dolutegravir resistance. J Antimicrob Chemother. 2019 Nov 1;74(11):3135–3149. 10.1093/jac/dkz256 31280314 PMC6798839

[5] Cevik M, Orkin C, Sax PE. Emergent resistance to dolutegravir among INSTI-naive patients on first-line or second-line antiretroviral therapy: A review of published cases. Open Forum Infect Dis. 2020;7(6):ofaa202. 10.1093/ofid/ofaa202 32587877 PMC7304932

[6] McCluskey SM, Pepperrell T, Hill A, Venter WD, Gupta RK, Siedner MJ. Adherence, resistance, and viral suppression on dolutegravir in sub-Saharan Africa: Implications for the TLD era. AIDS. 2021 Dec 15;35(Supplement 2):S127–S135. 10.1097/QAD.0000000000003082 34848579 PMC8647784

